# ﻿The arachnological collection at the Natural History Museum of Bern (NMBE), Switzerland: scope, history, and significance

**DOI:** 10.3897/zookeys.1258.167351

**Published:** 2025-11-03

**Authors:** Yvonne Kranz-Baltensperger, Christian Kropf, Manuela Sann

**Affiliations:** 1 Natural History Museum Bern, Bernastrasse 15, 3005 Bern, Switzerland Natural History Museum Bern Bern Switzerland; 2 University of Bern, Hochschulstrasse 6, 3012 Bern, Switzerland University of Bern Bern Switzerland; 3 Natural History Museum Basel, Augustinergasse 2, 4051 Basel, Switzerland Natural History Museum Basel Basel Switzerland

**Keywords:** Arachnida, digitization, museum collection, type material

## Abstract

The arachnological collection at the Natural History Museum Bern (NMBE) comprises approximately 120,000 vials containing around 500,000 specimens of spiders (Araneae), harvestmen (Opiliones), and scorpions (Scorpiones). While the material originated from around the globe, most of the specimens were collected in Austria, Germany, and Switzerland. The collection is organized into five main sections. It encompasses the collections of Konrad Thaler, Peter “Otto” Horak, Peter Sacher, and Ambros Hänggi, along with a general collection that features rare specimens, including material collected from caves. The material is stored in 80% pure ethanol. Since 1996, a total of 60,000 vials with 235,000 specimens of Araneae, representing 2080 different species, have been digitized and electronically catalogued. The collection also holds type material, including 25 holotypes and 453 paratypes of Araneae, three holotypes and paratypes of Opiliones, and one holotype and two paratypes of Scorpiones. A detailed list of the type material is provided in Suppl. material 1.

## ﻿Introduction

With their responsibility to store and maintain over decades of time specimens from all over the world, natural history museums play an important role as witnesses to the ever so vanishing diversity of organisms (Shaffer et al. 1998). In addition to curating, collection material is made available to researchers for a wide range of studies, including taxonomy, ecology, biogeography, or genetics. The data associated with the specimens can be used to reconstruct historical species distributions and community compositions, providing insights into species loss and ecosystem change (Drew 2011). Specimens often originate from rare or ecologically sensitive habitats, such as alpine zones, caves, peat bogs, and rainforest areas, or may have been collected from sites that are not accessible for field trips, often due to conservational, economic, or political constraints. Natural history collections therefore serve not only as repositories of biodiversity but also as essential contemporary witnesses and teaching facilities for students and taxonomists. Since the 1990s the digitization of collections has significantly enhanced accessibility, offering rapid access to a wide range of specimen data, including locality, collection date, high-resolution images, or sometimes additional information such as behavioral or habitat notes. A particular vital part of museum collections is the type material, which comprises irreplaceable reference specimens of exceptional scientific importance. This material must be carefully preserved for future generations, with their details systematically catalogued and made publicly available to facilitate research (Ballarin et al. 2020). In most museums, a significant proportion of the specimens has been acquired through donations or inheritances and remains only marginally identified, investigated, or digitized. Addressing these gaps requires the expertise of trained specialists, who can curate and prepare such material for scientific or faunistic research (Kielhorn 2024). Several initiatives have already successfully worked towards this goal. For example, the project of Planetary Biodiversity Inventory (2006), brought together researchers worldwide to identify, redescribe, and digitize oonopid spider specimens in museum collections. Similar, the SwissCollNet Project, initiated in 2022, aimed to enhance access to a wide range of Swiss museum collections, from botany and zoology to geology and anthropology. By following standardized protocols, these projects contributed valuable data for climate, biodiversity, and landscape research. The Natural History Museum in Bern (NMBE) houses a magnificent collection of terrestrial and aquatic invertebrates, including arachnids, insects, molluscs (gastropods and bivalves), and myriapods, primarily from Europe but also from other parts of the world. Despite the scientific value of these collections, particularly those of spiders, relatively little is known about their scope and composition in museums. The present study aims to address this gap.

## ﻿Material and methods

**Figure 1.**
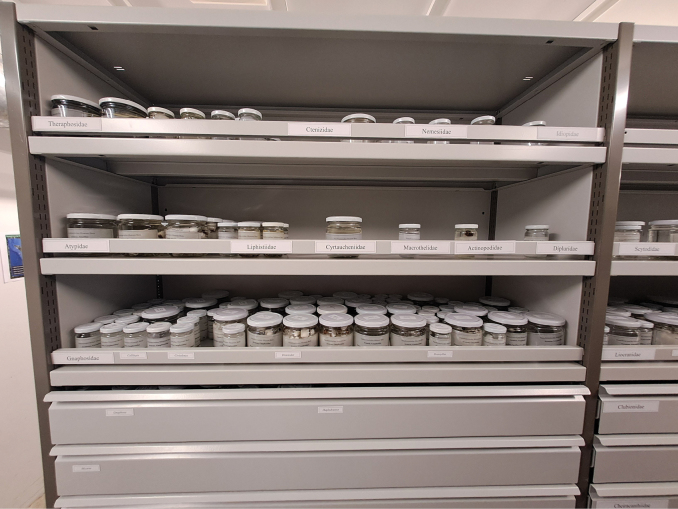
Example of the arachnological collection at the NMBE.

**Figure 2.**
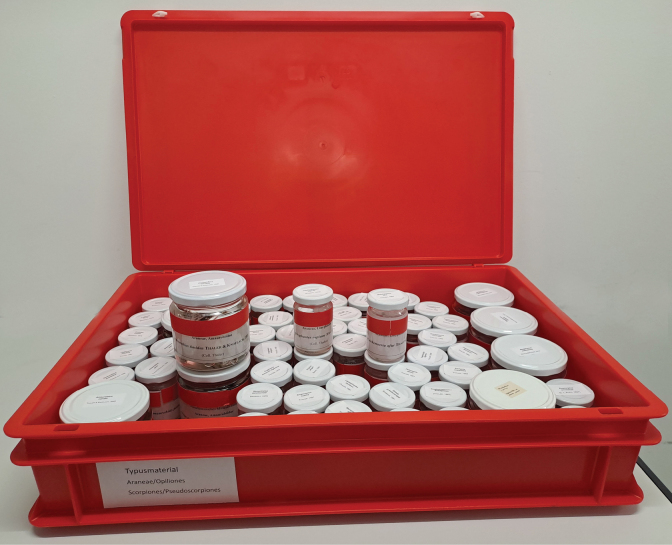
Type material of the NMBE arachnid collection. Red boxes ensure visibility for rapid evacuation.

The arachnological collection of the NMBE harbors individuals preserved in 80% pure ethanol (since 2011) and stored in the third basement level under controlled climatic conditions (35% relative humidity, 14 °C; Fig. 1). The type material (listed in the Suppl. material 1) is stored separately from the main collection to ensure rapid access for staff, emergency personnel, and fire service when needed (Fig. 2). Additionally, all information about the type material is regularly updated and available from the World Spider Catalog 2025; https://wsc.nmbe.ch/.

**Figure 3.**
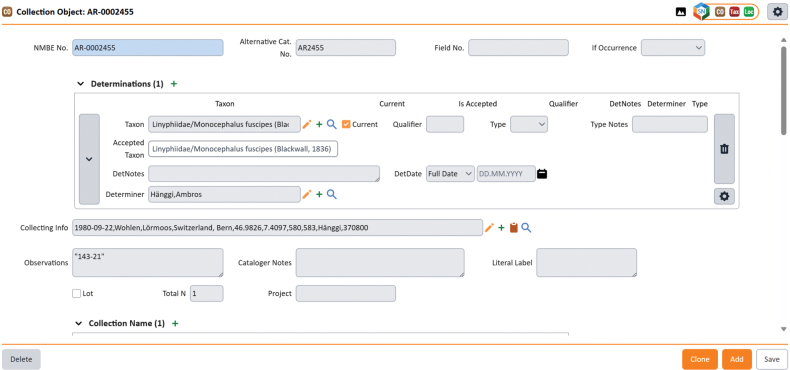
Screenshot from the Specify database. An example of a databased arachnid specimen is shown.

The arachnid collection is structured into five primary sections: the Thaler, Horak, Sacher, and Hänggi collections, along with a general collection, that also includes rare cave specimens from the Rohner collection. The Thaler and Horak collections were acquired through inheritance and remain only partially studied and digitized. In contrast, the Sacher collection is fully digitized and comprises specimens from various regions of eastern Germany. The Hänggi collection has been identified to species level, but is only partially digitized, while the general collection is almost completed. Each of the previous sections is described in detail below. Digitization of the arachnological collection began in 1993. Since then, arachnologists, students, and volunteers have systematically recorded available data into the Specify 7, Collection Management System, Specify Collections Consortium & Contributors, https://www.specifysoftware.org/ (Fig. 3). This includes specimen numbers, sex, collection locality and date, elevation, and other relevant information, as well as taxonomic identification to family, genus, species, and subspecies level, following the nomenclature of the World Spider Catalog version 26.

## ﻿Results

### ﻿The Thaler collection

The Thaler collection comprises arachnological material collected, preserved, and studied by Konrad Thaler and his students. Thaler (1940–2005) was a dedicated zoologist who made significant contributions to arachnology until his untimely death at the age of 64 during a field excursion in the Austrian Alps. Born in Innsbruck, Thaler studied zoology and botany at the University of Innsbruck, where he completed his doctoral thesis on the spider fauna of Northern Tirol. Over the course of his career, he became head of the department of terrestrial ecology and taxonomy at the same university, where he also earned his habilitation (Muster 2005). Further Thaler’s life has been described in various publications (e.g. Buchar 2007; Hauser 2007; Buchar and Merrett 2008). His scientific legacy includes not only an extensive collection of arachnids, diplopods, and insects but also numerous publications and articles, particularly on arachnid taxonomy, faunistics, and ecology (Knoflach 2009). Thaler supervised many student theses and is credited with the description of two new genera and 77 species of spider, primarily within the families Linyphiidae and Amaurobiidae, as well as one species of Opiliones (Muster 2005). Following his death, his collection was transferred to the NMBE in close collaboration with his wife Barbara Knoflach and his former students. It contains approximately 300,000 specimens, primarily from the Alps, collected between 1960 and 2005. Most of the material was identified by Thaler himself. The alpine spider specimens in particular present valuable opportunities for research in taxonomy, phylogenetics, and conservation biology, especially in the context of climate change and the vulnerability of alpine ecosystems. To date, 23,600 vials containing 114,258 spider specimens have been databased and made electronically accessible (accessed on January 2025). These specimens represent 58 families, with a predominance of Linyphiidae, Lycosidae, and Gnaphosidae, and originated mainly from Austria, Greece, Italy, and Switzerland. Many were collected from ecologically significant habitats such as peat bogs, swamps, caves, and alpine zones up to 3500 m in elevation. More than 48,000 vials containing around 200,000 spider specimens from the Thaler collection remain to be studied and digitized. In addition, numerous specimens of Opiliones and Diplopoda, many already identified, also form a part of Thaler’s scientific legacy. In total, the NMBE houses holotypes of 15 species and paratypes of 64 species in the Thaler collection, which are listed in Suppl. material 1.

### ﻿The Horak collection

Peter (“Otto”) Horak (1953–2015) was an Austrian arachnologist whose unexpected passing at the age of 62 marked the loss of a passionate and dedicated collector and spider enthusiast. His work focused primarily on the spider fauna of Styria, Austria, where he collected, studied, and meticulously documented arachnids and numerous other arthropods across a wide range of habitats. His deep knowledge of the region’s arthropod fauna led to numerous faunistic publications (Kropf and Komposch 2015). Horak also expanded his collection during travels to Australia, Africa, Costa Rica, and Greece. After his death, his extensive collection was transferred to the NMBE. To date, 9150 vials containing 28,064 specimens have been digitized and are electronically accessible. These specimens belong to 49 families, with a predominance of Lycosidae, Linyphiidae, and Agelenidae originating primarily from Austria, Australia, and Greece. Approximately two-thirds of the remaining material, consisting mainly of unidentified spiders from various European and African countries, has yet to be examined and determined. This collection also includes Opiliones, Scorpiones, and other invertebrates, along with an extensive personal library featuring historical works by Lister, Thorell, Menge, and others.

### ﻿The Sacher collection

The Sacher collection was transferred to the NMBE in 2016 and has been fully digitized. Peter Sacher, born in 1944 in Freiberg (Saxony), is a German arachnologist who has studied spiders since 1967, with a particular focus on the fauna and ecology of the families Araneidae and Tetragnathidae. His research centered on the faunistics and ecology of regions including Saxony, Saxony-Anhalt, Brandenburg, and Thuringia. Sacher has shown a strong interest in diverse and often rare habitats, such as dry meadows, dwarf-shrub heaths, swamps, and mountain streams. He is the author of 90 publications, primarily on spiders. His enormous knowledge in faunistics is reflected in his contributions to regional Red Lists of threatened species (Sacher 1993; Platen et al. 1998). Additionally, he edited the 1988 reprint of the “Monographie der Spinnen” written by Carl Wilhelm Hahn (Bauchhenss 2019), an important and previously inaccessible historical work in arachnology. The collection he donated to the NMBE includes 2818 vials containing 17,122 spider specimens, primarily from the families Linyphiidae, Lycosidae, and Thomisidae, along with 90 vials containing 408 specimens of Opiliones.

### ﻿The Hänggi collection

The Hänggi collection includes spider specimens collected by Ambros Hänggi during his undergraduate and doctoral research in the 1980s. His work focused on the spider fauna of various peat bog habitats in Wohlen and the Seeland region of Bern, Switzerland (Hänggi 1981–1982; Hänggi 1986). Approximately two-thirds of the collection has been digitized and is currently accessible, while the remaining third has been determined but is not yet fully sorted. This material holds particularly scientific value, as many of the habitats in which the specimens were collected have undergone significant ecological changes since the time of sampling (Hänggi and Maurer 1982).

### ﻿The general collection

The general collection primarily comprises spider specimens collected by Bodo von Broen, Max Bartels, Vygandas Relys, Andreas Rohner, and numerous others in the context of faunistic, ecological, and taxonomic studies. It also includes material gathered during the bachelor’s and doctoral research projects conducted by students at the NMBE. This part of the collection is almost fully digitized and accessible. Notably, it contains rare specimens from caves (collected by Andreas Rohner), as well as wetlands and alpine regions. In addition to spiders, the general collection houses scorpions collected by Adolf Scholl and Benjamin Gantenbein, mainly from North Africa and Switzerland, and various Opiliones specimens contributed by collectors from Austria, Germany, and Switzerland.

## ﻿Conclusions

The arachnological collection of the NMBE, with its extensive holdings of thoroughly investigated and electronically catalogued specimens of spiders, harvestmen, and scorpions, serves as a valuable resource, particularly for European researchers in the fields of taxonomy, faunistics, and genetics. In addition, the collection provides important data relevant to biogeography and historical species’ distribution. Many spiders are sensitive to changes in their habitat, for example as a result of climate change, urbanization, or agricultural practices (Branco and Cardoso 2020). Temporal comparisons of their presence or absence can provide important information about the state of an ecosystem and thus make them ideal bioindicators (e.g. Pearce and Venier 2006). As such, our museum collection holds significant potential for applied science, including the development of regional and national Red Lists. The unstudied material housed at the NMBE represents a further source of untapped scientific value. It likely contains numerous undescribed species, offering considerable opportunities for future taxonomic and biodiversity research.

## ﻿Additional information

### Use of AI

No use of AI was reported.

### Data availability

All of the data that support the findings of this study are available in the main text or Supplementary Information.

## Supplementary materials

Supplementary material 1 Comprehensive list of the type specimens hosted in the arachnological collection of the NMBE Data type xlsx https://binary.pensoft.net/file/1455435This dataset is made available under the Open Database License (http://opendatacommons.org/licenses/odbl/1.0/). The Open Database License (ODbL) is a license agreement intended to allow users to freely share, modify, and use this Dataset while maintaining this same freedom for others, provided that the original source and author(s) are credited.Yvonne Kranz-Baltensperger, Christian Kropf, Manuela Sann

## References

[B1] Ballarin F Salmaso R Latella L (2020) The arachnological collections of the Museo Civico die Storia Naturale of Verona (Italy) an overview. Arachnologische Mitteilungen / Arachnology Letters 60: 23–26. 10.30963/aramit6004

[B2] Bauchhenss E (2019) Geburtstag/Birthday. Zum 75. Geburtstag von Peter Sacher For the 75^th^ birthday of Peter Sacher. Arachnologische Mitteilungen 58: 5–6.

[B3] Branco V Cardoso P (2020) An expert-based assessment of global threats and conservation measures for spiders. Global Ecology and Conservation 24: e01290. 10.1016/j.gecco.2020.e01290

[B4] Buchar J (2007) In Erinnerung an meinen Freund Konrad Thaler, den unvergeßlichen Tiroler Arachnologen. Gredleriana 7: 395–398.

[B5] Buchar J Merrett P (2008) Konrad Thaler, 1940–2005. Bulletin - British Arachnological Society 14: 211–212. 10.13156/100.014.0405

[B6] Drew J (2011) The role of natural history institutions and bioinformatics in conservation biology. Conservation Biology : The Journal of the Society for Conservation Biology 25(6): 1250–1252. 10.1111/j.1523-1739.2011.01725.x 22070276

[B7] Hänggi A (1981–1982) Die Spinnenfauna des Lörmooses bei Bern. Lizentiatsarbeit.

[B8] Hänggi A (1986) Die epigäische Spinnenfauna der Feuchtgebiete des Grossen Mooses, Kt. Bern. Inaugural-Dissertation. Universitätsdruckerei Bern.

[B9] Hänggi A Maurer R (1982) Die Spinnenfauna des Lörmooses bei Bern—Ein Vergleich 1930/1980. Mitteilungen der Naturforschenden Gesellschaft in Bern 39: 159–183.

[B10] Hauser (2007) Epilog zum Nachruf von Jan Buchar auf Konrad Thaler. Gredleriana 7: 399–404.

[B11] Kielhorn KH (2024) Bemerkenswerte Spinnenfunde aus Sachsen-Anhalt—Teil IV (Arachnida: Araneae). Entomologische Mitteilungen Sachsen-Anhalt 32: 71–90.

[B12] Knoflach B (2009) Dokumente zum wissenschaftlichen Werk von Konrad Thaler. Contributions to Natural History 12: 1439–1567.

[B13] Kropf C Komposch C (2015) Nachruf/Obituary: In memoriam Dr. Peter “Otto” Horak, 29.5.1953–13.4.2015. Arachnologische Mitteilungen 50: 9–13.

[B14] Muster C (2005) Le temps marche si vite—Ein Nachruf auf Konrad Thaler. Arachnologische Mitteilungen 30: 1–12. 10.5431/aramit3001

[B15] Pearce JL Venier LA (2006) The use of ground beetles (Coleoptera: Carabidae) and spiders (Araneae) as bioindicators of sustainable forest management: a review. Ecological Indicators 6(4): 780–793. 10.1016/j.ecolind.2005.03.005

[B16] Planetary Biodiversity Inventory (2006) Planetary Biodiversity Inventory. [Accessed on 2025-09-09]

[B17] Platen R Blick T Sacher P Malten A (1998) Rote Liste der Webspinnen (Arachnida: Araneae) (Bearbeitungsstand: 1996, 2. Fassung) [List of endangered spider species (Red Data Book) (Arachnida: Araneae). Schriftenreihe für Landschaftspflege und Naturschutz 55: 268–275.

[B18] Sacher P (1993) Rote Liste der Webspinnen des Landes Sachsen-Anhalt. Berichte des Landesamtes für Umweltschutz, Sachsen-Anhalt 9: 9–12.

[B19] Shaffer HB Fisher RN Davidson C (1998) The role of natural history collections in documenting species declines. Trends in Ecology & Evolution 13(1): 27–30. 10.1016/S0169-5347(97)01177-4 21238186

[B20] World Spider Catalog (2025) World Spider Catalog. Version 26. Natural History Museum Bern. http://wsc.nmbe.ch [Accessed on 2025-09-09]

